# Hypoxia and classical activation limits *Mycobacterium tuberculosis* survival by Akt-dependent glycolytic shift in macrophages

**DOI:** 10.1038/cddiscovery.2016.22

**Published:** 2016-05-30

**Authors:** S K Matta, D Kumar

**Affiliations:** 1 Cellular Immunology Group, International Centre for Genetic Engineering and Biotechnology, Aruna Asaf Ali Marg, New Delhi 110067, India

## Abstract

Cellular reactive oxygen species (ROS) is a major antibacterial defense mechanism used by macrophages upon activation. Exposure of *Mycobacterium tuberculosis* (*Mtb*)-infected macrophages to hypoxia is known to compromise the survival of the pathogen. Here we report that the hypoxia-induced control of intracellular *Mtb* load in RAW 264.7 macrophages was mediated by regulating the cellular ROS levels. We show that similar to classical activation, hypoxia incubation of macrophages resulted in decreased mitochondrial outer membrane potential (MOMP) and a concomitant increase in the cellular ROS levels. Mitochondrial depolarization and consequently higher ROS could be blocked by knocking down Akt using siRNAs, which acted by inhibiting the switch to glycolytic mode of metabolism, an essential adaptive response upon classical activation or hypoxic incubation of macrophages. Moreover, in the classically activated macrophages or in the macrophages under hypoxia incubation, supplementation with additional glucose had similar effects as Akt knockdown. Interestingly, in both the cases, the reversal of phenotype was linked with the ability of the mitochondrial F_0_–F_1_ ATP synthase activity to maintain the MOMP in the absence of oxidative phosphorylation. Both Akt knockdown and glucose supplementation were also able to rescue *Mtb* survival in these macrophages upon classical activation or hypoxia incubation. These results provide a framework for better understanding of how the interplay between oxygen supply, which is limiting in the human tubercular granulomas, and nutrient availability could together direct the outcome of infections *in vivo*.

## Introduction

It is estimated that ~33% of the individuals are infected with *Mycobacterium tuberculosis* (*Mtb*),^1^ in which most of the infections persist in a latent state throughout life. *Mtb* have the ability to persist in a metabolically dormant state for years, with antibiotic tolerance.^2^ Latent *Mtb* reside in the infected tissue lesions called granulomas, which is surrounded by immune cells with accumulation of caseous and necrotic debris in its center.^3^ The oxygen tension in the granulomas is low and comparable to that of the tumors.^4^ It has been shown that macrophages shifts toward glycolytic metabolism and survive better under less oxygen tension.^5^ Macrophages under hypoxia also increase their ability to decrease intracellular load of *Mtb* by secretion of *β*-defensin-2 and activation of vitamin D receptor pathway.^6^ Similar to hypoxia, classical activation of macrophages also shift metabolism toward glycolysis.^7^ This phenomenon of glycolytic shift is also required for the activation of other immune cells specifically with pro-inflammatory phenotypes, such as Th1, Th17 and dendritic cells (DC).^8^ Classical activation of macrophages with interferon gamma (IFN-*γ*) and/or lipopolysaccharide (LPS) treatment increases their oxidative potential along with acidification of the lysosomal compartments, which are involved in the pathogen clearance.^9^

Aerobic glycolysis is a hallmark of Warburg effect, in which cancer cells preferentially convert glucose to lactate and inhibit oxidative phosphorylation.^10^ HIF-1*α* (hypoxia inducible factor-1 alpha, a transcription factor) and mTOR (mammalian target of rapamycin, a metabolic kinase) are now recognized as the key regulators of this shift of metabolism toward glycolysis.^11^ Recently, the regulation of glycolytic shift in metabolism by Akt-mTOR/HIF-1*α* signaling was elucidated with respect to trained immunity in macrophages.^12^ It was shown that glycolytic shift of metabolism upon LPS stimulation of *β*-glucan-primed macrophages was dependent on HIF-1*α*-mediated upregulation of genes pertaining to glycolysis and mTOR pathway. Apart from HIF-1*α*, Akt is also known to stimulate aerobic glycolysis in cancer cells.^13^ Further, Akt has been shown to modulate macrophage inflammatory response to *Francisella* infection.^14^ Interestingly, cellular reactive oxygen species (ROS) resulted from M-CSF activation of macrophages stimulates Akt phosphorylation.^15^ ROS is a key innate defense mechanism against intracellular pathogens. Although increase in ROS upon classical activation of macrophages is understood as an antibacterial mechanism,^9^ their existence under hypoxic environment is still not clear. However, there are observations in the literature, which supports the presence of ROS under hypoxia^16–18^ and the possible mechanism of its generation.^19^ ROS production increases upon inhibition of the electron transport chain (ETC).^20^ Hypoxia is known to inhibit the ETC owing to the lack of oxygen as terminal electron acceptor.^21^

Here we report that hypoxic incubation of macrophages leads to decrease in intracellular *Mtb* load similar to that of classical activation of macrophages. We show that the microbicidal potential of cells under hypoxia derives from a seemingly similar set of mechanisms as in the case of classical activation. We also show a strong correlation between the rescue of macrophage survival with that of *Mtb* under these conditions.

## Results

### Glycolytic shift and *Mtb* survival in RAW 264.7 macrophages under hypoxia

**Figure 1:**
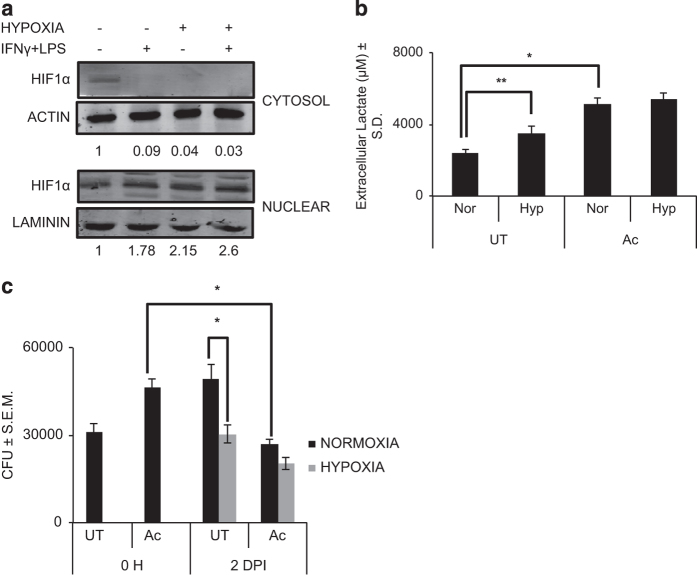
Glycolytic shift and *Mtb* survival in RAW 264.7 macrophages under hypoxia. (**a**) HIF-1*α* immunoblot for nuclear and cytosolic extracts of RAW 264.7 cells with and without 48 h of IFN-*γ*+LPS (IFN-*γ* (100 U/ml) and LPS (20 ng/ml) for 48 h) treatment and hypoxic (0.5% O_2_) incubation. Actin and laminin were used as loading controls for cytosolic and nuclear extracts, respectively. (**b**) Extracellular lactate concentration of untreated (UT) and IFN-*γ*+LPS-activated (Ac) RAW 264.7 cells under normoxic (Nor, 21% O_2_) and hypoxic (Hyp, 0.5% O_2_) incubation for 48 h. *Y* axis shows average±S.D. of at least three independent sets of experiments performed in triplicates. (**c**) CFU assay showing the *Mtb* (H37Rv) CFU for untreated (UT) and activated (Ac) RAW 264.7 cells kept under normoxia and hypoxia at 0 h post infection (0 H) and 2 DPI. *Y* axis shows average±S.E.M. of at least three independent sets of experiments performed in triplicates. * and ** denotes significant difference between compared sets at *P*<0.01 and *P*<0.05 respectively using Student’s *t*-test.

HIF-1*α* stabilization acts as one of the markers of glycolytic shift in metabolism, as it acts as a transcriptional factor for upregulating the expression of glycolytic genes.^22^ Therefore, HIF-1*α* stabilization of RAW 264.7 cells was evaluated as a marker for glycolytic shift. The cells were classically activated (100 U/ml of IFN-*γ* and 20 ng/ml of LPS) or kept untreated under normoxia and hypoxia for 48 h. Expectedly, there was a significant increase in the HIF-1*α* nuclear levels in hypoxia-incubated RAW 264.7 cells (Figure 1a). Nuclear levels of HIF-1*α* also increased in classically activated macrophages under both normoxia and hypoxia (Figure 1a). However, the cytosolic levels of HIF-1*α* reduced in RAW 264.7 macrophages upon classical activation or hypoxic incubation for 48 h (Figure 1a). This clearly suggested a net translocation of the HIF-1*α* transcription factor from the cytosol to the nucleus upon classical activation or hypoxic incubation of macrophages, which is a common phenomenon for many other cell types upon hypoxic incubation.^23^ Consequentially, extracellular lactate levels were determined to confirm the glycolytic shift of metabolism. As expected, RAW 264.7 cells showed a significant increase in the extracellular lactate levels upon exposure to hypoxia and classical activation (Figure 1b). However, lactate accumulation in the control cells under hypoxia was significantly lower than classically activated cells under normoxia or hypoxia. Second, there was no difference in lactate accumulation in classically activated macrophages under normoxia or hypoxia. These observations further suggested that the response of shift to glycolysis was profound upon classical activation than hypoxic incubation of macrophages, leading to enhanced accumulation of lactate eventually. This response upon classical activation was also independent of the O_2_ levels. In addition to a glycolytic shift in metabolism, both classical activation and hypoxia incubation are known to increase the microbicidal potential in the macrophages. We then evaluated their microbicidal potential against the intracellular pathogen *Mtb*. Activated cells were much more efficient in taking up *Mtb*, as immediately after infection they showed much higher bacterial load (colony forming unit (CFU)) compared to untreated control (Figure 1c). Activated cells were also more efficient in killing intracellular *Mtb* than untreated macrophages at 2 days post infection (DPI) under normoxia (Figure 1c). Intracellular survival of *Mtb* was significantly inhibited in hypoxia incubated untreated cells (Figure 1c). Interestingly, activated cells under hypoxia were equally efficient in killing *Mtb* as in normoxia (Figure 1c).

### Antibacterial responses upon hypoxic incubation and classical activation of macrophages

**Figure 2:**
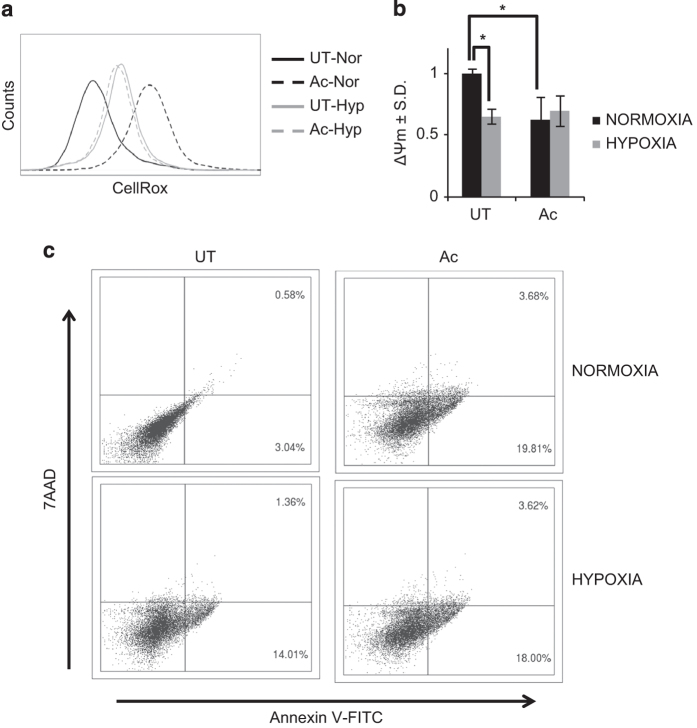
Antibacterial responses upon hypoxic incubation and classical activation of macrophages. (**a**) Line histograms of 10 000 untreated (UT) and activated (Ac) cells under normoxic (Nor) and hypoxic (Hyp) incubation for 48 h, stained with CellROX Green to measure cellular ROS levels. (**b**) JC-1 ratio (MOMP (ΔΨ_m_)): JC-1 emission at 625 nm (JC-1 aggregates) to JC-1 emission at 535 nm (JC-1 monomers) upon 488 nm excitation for untreated (UT) and activated (Ac) RAW 264.7 cells under normoxia and hypoxia for 48 h. *Y* axis shows average±S.E.M. of at least three independent sets of experiments performed in triplicates. * denote significant difference between compared sets at *P*<0.01 using Student’s *t*-test. (**c**) Scatterplots for Annexin-V-FITC- and 7-AAD-stained untreated (UT) and activated (Ac) cells under normoxia and hypoxia for 48 h with respective percentage of cells in their quadrants.

Apart from glycolytic shift, classical activation of macrophages also leads to increase in many of the pathogen-clearing mechanisms, including high redox potential.^9^ Therefore, it was imperative to measure the similarity in antibacterial mechanisms upon hypoxic incubation to that of classical activation of macrophages. Cellular ROS levels were significantly increased upon hypoxic incubation of macrophages (Figure 2a). Similar to lactate accumulation, the increase in ROS under hypoxia was less compared to that in classically activated macrophages (Figure 2a). However, under hypoxia there was no further increase in the levels of cellular ROS upon classical activation (Figure 2a). ETC is the major source of cellular ROS, especially when it is inhibited.^24,25^ ETC is inherently inhibited under hypoxia due to lack of O_2_ as the terminal acceptor for electron, whereas in the activated macrophages, nitric oxide production inhibits complex I of the ETC.^21,26^ To note, classical activation under hypoxia cannot induce nitric oxide production, as that requires molecular oxygen. Thus, ROS generated from ETC is considered as a marker for the inhibition of oxidative phosphorylation and the loss of mitochondrial potential.^27^ Therefore, mitochondrial outer membrane potential (MOMP) of cells was also evaluated post 48 h of hypoxic incubation and classical activation using JC-1 dye. Both hypoxic incubation and classical activation of macrophages led to a significant decrease in the MOMP (ΔΨ_m_, Figure 2b). The loss of MOMP and higher ROS are known to cause cell death.^28^ We have shown previously the high degree of cell death in RAW 264.7 macrophages upon IFN-*γ*+LPS treatment under normal O_2_ levels.^29^ There was an increase in cell death upon hypoxic incubation of macrophages for 48 h (Figure 2c). Classical activation of macrophages caused comparable cell death under both normoxia and hypoxia, which was relatively higher than hypoxia incubation alone (Figure 2c). Apoptosis is one of the general defense mechanism of macrophages against intracellular *Mtb*.^30^ Therefore, it was now clear that macrophages acquire antibacterial mechanism similar to classically activated macrophages under hypoxic incubation.

### Akt mediates glycolytic shift and maintains cellular ROS

**Figure 3:**
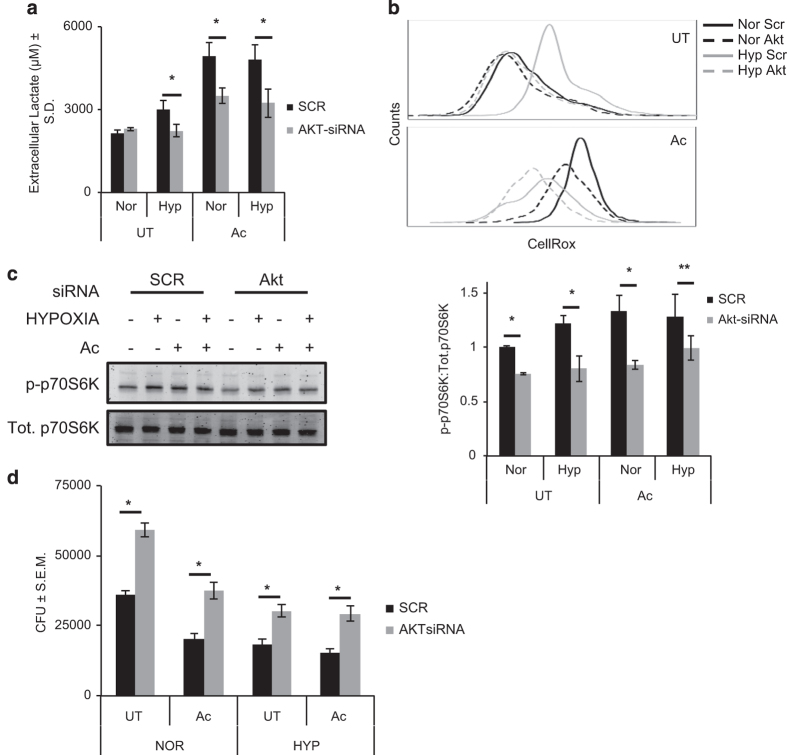
Akt mediates glycolytic shift and maintains cellular ROS. (**a**) Extracellular lactate levels for untreated (UT) and activated (Ac) RAW 264.7 cells treated with 50 nM of SCR or Akt-siRNA for 48 h kept under normoxia and hypoxia. *Y* axis shows the average±S.D. of three independent experiments. (**b**) Line histograms of 10 000 untreated (UT) and activated cells with 50 nM of SCR or Akt-siRNA for 48 h under normoxia (Nor) and hypoxia (Hyp) stained with CellROX Green to measure cellular ROS levels. (**c**) Immunoblot for p-p70S6K and total p70S6K (Tot.) for untreated (UT) and activated (Ac) RAW 264.7 cells with 50 nM of SCR or Akt-siRNA for 48 h under normoxia (Nor) and hypoxia (Hyp). Ratio of p-p70S6K to total p70S6K normalized to UT cells under normoxia was used to calculate mTOR activity. *Y* axis shows average±S.E.M. of at least three independent sets of experiments. (**d**) *Mtb* (H37Rv) CFU for untreated (UT) and activated (Ac) RAW 264.7 cells kept under normoxia and hypoxia at 48 h post infection treated along with 50 nM of scrambled (SCR) or Akt-siRNA. *Y* axis shows average±S.E.M. of at least three independent sets of experiments performed in triplicates. For **a**, **b** and **d** * and ** denote significant difference between compared sets at *P*<0.01 and *P*<0.05 using Student’s *t*-test.

mTOR and Akt are cellular kinases that act as hubs of cellular signaling, controlling cellular metabolism, development, protein synthesis, survival and so on.^31^ Akt also acts as a major regulator of mTOR activity.^32^ Recently it was found that Akt-mTOR/HIF-1*α* signaling axis controls glycolytic shift of metabolism in macrophages with respect to trained immunity.^12^ Apart from mTOR-mediated control of glycolytic shift, Akt is also known to induce glycolytic shift in DC mediated by toll-like receptor-induced signaling.^33^ Previously we reported inhibition of glycolytic shift in classically activated macrophages upon Akt knockdown.^29^ Therefore, we measured the effect of Akt knockdown on hypoxia- and activation-induced glycolytic shift. Akt knockdown by siRNA resulted in a significantly lowered lactate accumulation in the macrophages under hypoxia (Figure 3a). Moreover, lactate accumulation was also significantly lower in the activated cells under both normoxia and hypoxia upon Akt knockdown (Figure 3a). It clearly suggested that Akt was involved with the glycolytic shift in these macrophages. We confirmed, by western blot that there was a significant knockdown of Akt post 48 h of incubation with Akt-siRNA in RAW 264.7 macrophages (Supplementary Figure S1). Following this, cellular ROS levels were also measured upon Akt knockdown to ascertain the effect of glycolytic shift on activation phenotype of macrophages. Expectedly, cellular ROS levels were significantly reduced upon Akt knockdown under hypoxia (Figure 3b). Moreover Akt knockdown also brought down ROS in activated macrophages under both normoxia and hypoxia (Figure 3b). It clearly indicated that Akt-mediated signaling was responsible for maintaining cellular ROS along with glycolytic shift in metabolism, a phenotype we previously reported for classically activated macrophages.^29^ mTOR activity (p-p70S6K T389) was also measured as a marker for upregulation of signaling associated with Akt-mediated glycolytic shift. Control RAW 264.7 macrophages under hypoxia or activated cells under normoxia or hypoxia showed much higher p-p70S6K levels compared to untreated control cells under normoxia (Figure 3c). Akt knockdown led to a significant decrease in the p-p70S6K levels in control and activated RAW 264.7 macrophages under both normoxia and hypoxia (Figure 3c). It indicated that the induction of Akt/mTOR signaling upon classical activation is independent of O_2_ levels. As Akt-mTOR signaling axis forms the basis of metabolic shift to glycolysis in activated macrophages, the effect of Akt knockdown was then monitored to evaluate the significance of this kinase in regulating intracellular H37Rv survival. Akt knockdown led to a significant increase in *Mtb* CFU in both untreated and classically activated cells under normoxia and hypoxia 2 DPI (Figure 3d).

### Mitochondrial depolarization is key to hypoxia- and activation-induced phenotypes

**Figure 4:**
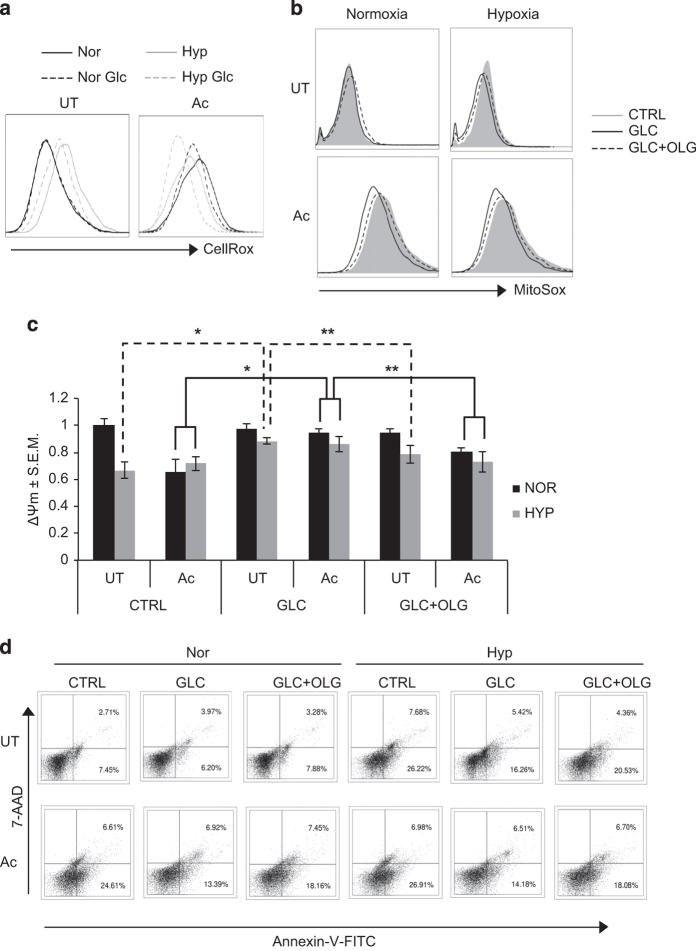
Mitochondrial depolarization is key to hypoxia- and activation-induced phenotypes. (**a**) Line histograms of 10 000 untreated (UT) and activated (Ac) cells under normoxic (Nor) and hypoxic (Hyp) incubation for 48 h with and without glucose supplementation at 24 h (Glc), stained with CellROX Green to measure cellular ROS levels. (**b**) Line histograms of cells stained with MitoSOX, (**c**) JC-1 ratio, (**d**) scatterplots for Annexin-V-FITC- and 7-AAD-stained H37Rv-infected cells. The cells were incubated with solvent control (CTRL) and glucose (10 mM, GLC) without and with oligomycin (200 nM, GLC+OLG) post 24 h of incubation under normoxia and hypoxia for 48 h. * and ** denote significant difference between compared sets at *P*<0.01 and *P*<0.05 using Student’s *t*-test.

As Akt knockdown resulted in a decrease in the glycolytic flux, we next wanted to test whether the effect of hypoxia or activation was due to glucose depletion from the media as a consequence of high glycolytic rate. In classically activated macrophages, we previously showed Akt-mediated effects on cellular ROS and apoptosis were dependent on the availability of glucose in the media.^29^ We therefore next monitored the effect of glucose supplementation on RAW 264.7 macrophages under hypoxic incubation or upon activation. ROS levels upon glucose supplementation under these conditions followed the pattern similar to that of ROS upon Akt knockdown (Figure 4a). ROS levels were decreased response in activated cells under both normoxia and hypoxia upon glucose supplementation (Figure 4a). Mitochondrial superoxide generation was also measured as one of the possible sources of cellular ROS, as mitochondrial ROS has been shown as bactericidal in the activated macrophages.^34^ MitoSOX was used to determine mitochondrial superoxide levels inside the cells. Similar to cellular ROS, mitochondrial superoxide levels were also decreased upon glucose supplementation in activated cells under both normoxia and hypoxia (Figure 4b). There was also a substantial decrease in MitoSOX staining of control cells upon glucose supplementation under hypoxia (Figure 4b). It is known that in cells relying mostly on the glycolytic metabolism, mitochondrial potential is maintained by reversal of mitochondrial ATP synthase activity by utilizing glycolytic ATP.^35^ To test whether the decline in cellular ROS and mitochondrial superoxide upon glucose supplementation was a result of utilization of glycolytic ATP to maintain MOMP, we used oligomycin, a specific inhibitor of F_0_–F_1_ ATP synthase. In the presence of oligomycin (200 nM), the rescue of mitochondrial superoxide production by glucose supplementation was abolished (Figure 4b). Similar effect was observed in the levels of cellular ROS (Supplementary Figure S2). Next we monitored the MOMP using JC-1 stain. Under hypoxia or upon classical activation, as shown earlier (Figure 2b), there was a decline in the MOMP. Glucose supplementation was able to rescue the MOMP under each of these conditions (Figure 4c). However, the effect of glucose supplementation was significantly reduced when cells were also treated with oligomycin (Figure 4c). Glucose supplementation to activated macrophages under normoxia or hypoxia, as well as to non-activated cells under hypoxia rescued them from cell death (Figure 4d). In line with the observations above, glucose supplementation in the presence of oligomycin failed to rescue the cells from death (Figure 4d). Akt knockdown, which also inhibited shift to glycolysis in the activated or hypoxia-incubated macrophages, resulted in similar rescue in the survival of *Mtb-*infected cells (Supplementary Figure S3).

### Mitochondrial depolarization is central to the increased microbicidal ability of macrophages under hypoxia or upon activation

**Figure 5:**
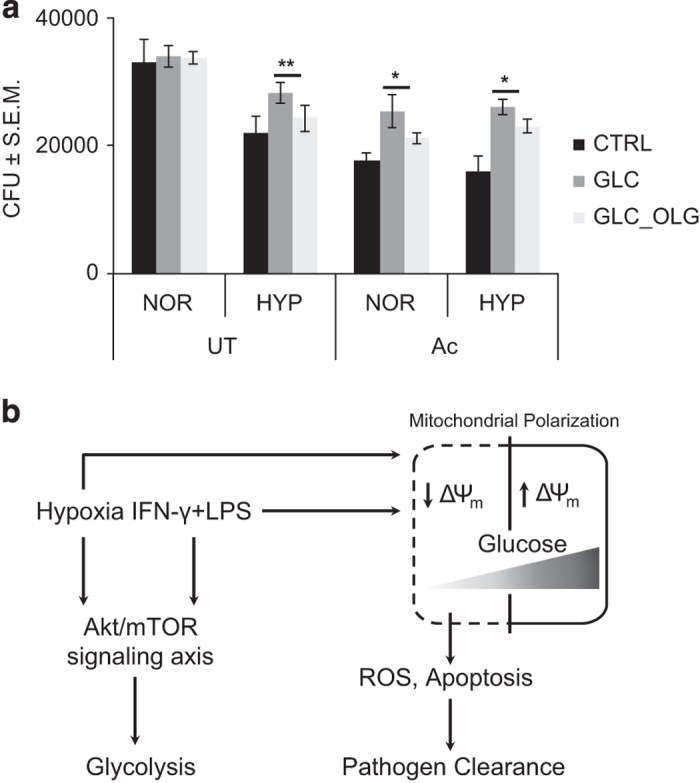
Mitochondrial depolarization is central to the increased microbicidal ability of macrophages under hypoxia or upon activation. (**a**) *Mtb* (H37Rv) CFU for untreated (UT) and activated cells (Ac) under 48 h of normoxia (NOR) and hypoxia (HYP). The cells were incubated with solvent control (CTRL) and glucose (10 mM, GLC) without and with oligomycin (200 nM, GLC_OLG) post 24 h of incubation under normoxia and hypoxia for 48 h. * and ** denote significant difference between compared sets at *P*<0.01 and *P*<0.05 using Student’s *t*-test. (**b**) Model for Akt-regulated glycolytic shift in controlling intracellular *Mtb* clearance upon hypoxic incubation and classical activation of macrophages.

As shown above, glucose supplementation acted in a manner that was similar to the condition when Akt was knocked down using siRNA. As Akt knockdown also rescued *Mtb* survival, we next tested whether glucose supplementation could also help in bacterial survival. Expectedly, glucose supplementation at 24 h in activated cells significantly increased *Mtb* CFU at 2 DPI (Figure 5a). There was also a significant increase of *Mtb* CFU in non-activated cells upon glucose supplementation under hypoxia (Figure 5a). However, there was no change in *Mtb* CFU to glucose supplementation in the control cells under normoxia. As oligomycin desensitized the cells under each of the conditions studied here to respond to glucose supplementation, we tested its effect on *Mtb* CFU as well. The increase in the *Mtb* CFU upon glucose supplementation in the non-activated hypoxia-incubated macrophages or in the activated macrophages under either normoxia or hypoxia was compromised in the presence of oligomycin significantly (Figure 5a).

## Discussion

Cellular ROS production is considered as one of the major bactericidal mechanisms by the innate immune cells such as the macrophages and DC upon intracellular infections. In addition, shift in the metabolism toward glycolysis is now much more recognized as a limiting step in order to gain effector functions of immune cells such as macrophages, DC and T cells.^36,37^ This study provides evidence for inverse correlation between cellular ROS levels and glucose levels in the cells that rely majorly on the glycolytic metabolism for survival. The study suggests that classical activation and hypoxic incubation of macrophages make them dependent on glycolysis for their survival, which in turn leads to induction of many bactericidal mechanisms such as increase in cellular ROS and apoptosis.

Stabilization of HIF-1*α* and an active mTOR signaling was observed upon classical activation and hypoxic incubation of macrophages. HIF-1*α* is known as a marker for adaptation of cells to hypoxia for survival by transcriptionally upregulating the expression of glycolytic genes.^22^ Moreover, mTOR along with HIF-1*α* signaling has been recently shown to form the basis of glycolytic shift in murine macrophages.^12^ Therefore, shift in the metabolism to glycolysis was expected in macrophages upon classical activation and hypoxic incubation. We also observed increase in cellular ROS and induction of apoptosis upon both hypoxic incubation and classical activation of macrophages, which correlated with the intracellular survival of *Mtb* under these phenotypes. To establish the direct correlation between glycolytic shift and increased microbicidal activity in these macrophages, we knocked down Akt using siRNAs. Akt is known to regulate the shift to glycolytic mode of metabolism.^33^ Akt knockdown expectedly brought down cellular ROS levels and glycolytic flux. In a surprising result, Akt knockdown also helped increased survival of *Mtb* in the activated and/or hypoxia-incubated macrophages. Thus, in the context of activated macrophages, Akt acts more as an antibacterial host factor most likely by regulating the cell death pathway. Akt is a well-known pro-survival molecule, which prevents cell death under signals and conditions inducing apoptosis. Akt has been shown to promote glucose utilization to inhibit conformational change in Bax and storage of glucose as glycogen via inhibition of glycogen synthase kinase-3.^38,39^ However, there are also evidences that suggests that Akt can act as a pro-apoptotic molecule under many conditions.^40^ Akt has been shown to be dependent on glycolysis and glucose for its pro-survival functioning like suppression of Puma and Mcl-1 synthesis.^41,42^ In the classically activated macrophages under normoxia, we previously showed a pro-apoptotic role of Akt.^29^ Prevention of apoptosis by Akt knockdown in activated macrophages or macrophages exposed to hypoxia—both undergone a shift in the metabolism to glycolysis, also supported the pro-apoptotic function of Akt in macrophages in the context of classical activation and hypoxic incubation. Similar rescue of cell death by glucose supplementation corroborated the dependency of Akt on glucose to maintain its more acknowledged pro-survival function.

Similar pattern of cellular ROS and mitochondrial ROS levels in control and glucose supplemented cells under glycolytic shift suggests the role of glucose in regulating mitochondrial ROS. It is important to note here that glucose availability helps maintain the MOMP in the cells that are dependent on glycolytic metabolism.^35^ Similar levels of intracellular *Mtb* upon Akt knockdown and glucose supplementation strongly suggested that bacterial clearing capacity of macrophages is directly proportional to mitochondria-derived ROS inside the cell, which in turn is dependent on differential glucose utilization. This correlation of increased mitochondrial ROS with intracellular *Mtb* clearance was further corroborated by the use of oligomycin, which inhibits glycolysis-mediated maintenance of mitochondrial potential and prevents the rescue of cell death in activated macrophages upon glucose supplementation. This observation further confirms the role of glycolytic ATP in maintaining the MOMP through regulating ATP synthase activity. Macrophages with reduced MOMP were also associated with increased mitochondrial ROS and cell death along with better capacity to limit intracellular *Mtb* survival.

In classically activated macrophages, we have shown previously, shift to glycolysis was accompanied with a decline in cellular autophagy leading to the accumulation of depolarized mitochondria and higher cellular ROS followed by cell death.^29^ We also showed that this phenotype could be reverted by either Akt knockdown or supplementation with glucose.^29^ It will be interesting to observe how macrophages respond to hypoxia in terms of autophagy regulation. In other cellular systems, incubation under hypoxia is believed to induce autophagy.^43^ However, the phenotypes observed in this study, such as increased cellular ROS and lowered MOMP suggest otherwise. Interestingly, it would also mean that in the activated macrophages or hypoxia-incubated macrophages, restoration of autophagy by Akt knockdown or glucose supplementation could rescue *Mtb* survival. This proposition however seems contrary to the existing understanding on regulation of *Mtb* infection by autophagy^44^ and therefore requires much more focused study for a clear perspective. In this context it is important to mention our recent findings where we show virulent *Mtb* could selectively inhibit only xenophagy flux without disturbing the basal cellular autophagy.^45^ This selectivity may help it evade killing and rather survive better under the conditions where macrophages are rescued from cell death by inducing autophagy.

Physiologically, this study provides an interesting insight into the interplay of various factors regulating the outcome of *Mtb* infection in the hosts. The tubercular granuloma, formed as a result of immune control of infection, is known to be hypoxic at the core. Many macrophages infected with the bacilli remain closer to the core of the granuloma, which are further surrounded by neutrophils, lymphocytes and epithelioid cells.^46^ This study suggests that the activation of inflammatory pathways, as well as sequestering the *Mtb* to the hypoxic environment may not yield the desired outcome of bacterial clearance as long as nutrient availability to the core is not curtailed.

In conclusion, classical activation and hypoxic incubation of macrophages leads to increased bactericidal responses such as cellular ROS production and apoptosis (Figure 5b). Glycolytic shift in metabolism under both conditions is regulated by Akt-mTOR signaling axis. In the *in vitro* settings, increased glycolytic shift results in glucose depletion in the media and consequent decline in glycolytic ATP production to maintain the MOMP, which eventually results in cell death. Macrophages in this state acquire increased microbicidal potential, mostly through increased cellular ROS and resulting cell death. Inhibition of glycolytic shift by knocking down Akt or maintaining a constant supply of glucose to the extracellular media allows prolonged MOMP maintenance even in the absence of oxidative phosphorylation. Depletion of Akt or glucose supplementation rescues *Mtb* survival under hypoxia or in activated macrophages under both normoxia and hypoxia.

## Materials and Methods

### Reagents and antibodies

7-AAD and Annexin-V-FITC were from Cayman Chemical (Ann Arbor, MI, USA). Mouse IFN-*γ* was from eBiosciences (San Diego, CA, USA). LPS and oligomycin were from Sigma Aldrich (St. Louis, MO, USA). JC-1, MitoSOX and CellROX Green were from Molecular Probes, Life Technologies Corporations (Grand Island, NY, USA). Rabbit polyclonal anti-phospho-p70S6K-T389, laminin, HIF-1alpha, phospho-Akt-S473 and p70S6K were from Cell Signaling Technologies (Danvers, MA, USA). Goat polyclonal anti-Akt and rabbit polyclonal anti-Actin were from Santacruz Biotechnologies (Dallas, TX, USA). Goat anti-Rabbit IgG-IRDye 800CW and Donkey anti-Goat IgG-IRDye 800CW were from LI-COR BioSciences (Lincoln, NE, USA).

### Cell culture and classical activation of macrophages

RAW 264.7 murine macrophage cells were cultured at 37 °C and 5% CO_2_ in high glucose-containing Dulbecco’s modified Eagle’s media (DMEM, Life Technologies Corporations) with 10% fetal bovine serum (FBS, Life Technologies Corporations). For hypoxia experiments, the complete medium (10% FBS v/v in DMEM) was pre-equilibrated at 37 °C, 5% CO_2_ and 0.5% O_2_ for 24 h before using it for incubation with cells under hypoxic conditions. For classical activation, RAW 264.7 cells were treated with IFN-*γ* (100 U/ml, of specific activity of ~1 U/ng) and LPS (20 ng/ml) for 24 h. Cells were then washed with warm DMEM once and kept in complete medium as per experimental setup. For glucose supplementation experiments, glucose was added at final concentration of 10 mM 24 h before harvesting for time points at 48 h. Oligomycin was added into the culture medium 24 h before harvesting at 48 h time point. For experiments pertaining to hypoxia, all the solutions for varied treatments were kept along with the experimental samples in hypoxia to get equilibrated in the hypoxic environment for their addition at 24 h before harvesting at 48 h.

### Bacterial culture and CFU assays

*Mtb* H37Rv strain was a kind gift from K.V. Rao. *Mtb* cultures were grown in 7H9 broth (BD Holdings Pte Ltd, Singapore) with 10% v/v ADC, 0.5% v/v glycerol and 0.05% v/v Tween-80. For CFU experiments, 2×10^4^ untreated or activated cells were plated into each well of a 96-well plate. Cells were infected with *Mtb* at a MOI of 10 following the protocol described previously.^47^ At the requisite time points, the macrophages were lysed in 0.06% w/v SDS in 7H9, and plated onto the 7H11 agar (BD Holdings Pte Ltd) plates with 10% v/v OADC. The plating was performed using the 10-fold serial dilutions of the lysates in 7H9 medium, and colonies were counted post 10–12 days of incubation at 37 °C.

### Cell lysis and immunoblotting

For immunoblotting experiments, 1.5×10^6^ cells were plated per well of a six-well plate. Cells were washed twice with ice cold DMEM before their incubation with buffer A (20 mM HEPES, 10 mM NaCl, 1.5 mM MgCl_2_, 0.2 mM EDTA and 0.5%v/v Triton-X-100) with 1× Protease Arrest (G-Biosciences, St. Louis, MO, USA) for 20 min on ice for lysis. The lysates were centrifuged at 4 °C at 6000 *g* for 10 min and the supernatant was collected. For nuclear and cytosolic extracts, the cells were processed as per NE-PER Nuclear Extraction Kit’s (Thermo Scientific, Waltham, MA, USA) instructions. Protein concentration in the supernatant was quantified using Bradford’s reagent (Bio-Rad, Hercules, CA, USA) using BSA as standard. The lysates were subjected to SDS-PAGE and transferred onto the nitrocellulose membrane for immunoblotting. The blots were incubated with blocking solution (Odyssey buffer (LI-COR Biosciences) in 1:1 dilution with 1× PBS) at room temperature for 2 h. The blots were then immunoblotted with primary antibodies followed by secondary antibodies made in blocking solution with in between washings with 1× PBST. The infrared imaging of the blots was done using the Odyssey Infra red Imaging system (LI-COR Biosciences).

### Flow cytometry and fluorimetry

For flow cytometric measurements of cellular ROS, MOMP and cell death distribution, 2×10^5^ cells were plated per well in a 24-well plate. The cells were scrapped at requisite time points and stained with the following reagents before acquisition of the parameters using BD FACSDiva acquisition software in BD FACS Canto II flow cytometer. Cellular ROS was measured using CellROX Green, MOMP using JC-1, mitochondrial superoxide using MitoSOX and cell death distributions using Annexin-V-FITC and 7-AAD. The time and staining concentrations of all the fluorescent dyes were performed as per the manufacturer’s directions. The data were analyzed and plotted using the R-packages ‘flowCore’^48^ and ‘flowViz’.^49^ For measurement of MOMP in hypoxia experiments, 2×10^4^ cells were plated per well in a 96-well plate. The staining buffer of JC-1 staining (Life Technologies Corporations) was kept in the hypoxic conditions along with experimental samples to get equilibrated for its use at the 48 h time point. The staining was performed as per the manufacturer’s direction and the plates were sealed extensively with parafilms before measurement of fluorescence emission at 535 (JC-1 monomers) and 600 nm (JC-1 aggregates) with excitation at 488 nm, using Perkin Elmer VICTOR3 1420 Multilabel Plate Reader (PerkinElmer, Inc., Waltham, MA, USA). The ratio of JC-1 aggregates to JC-1 monomers was plotted after normalization to the untreated normoxia control to depict changes in MOMP (ΔΨ_m_).

### Measurement of lactate

For lactate measurements, 2×10^4^ cells were plated per well in a 96-well plate. Cells were maintained in 1% v/v FBS in DMEM for the entire length of experiment, as per the directions of the kit. Extracellular lactate was measured using lactate measurement kit following the manufacturer’s protocol (Cayman Chemical).

### Statistical analysis

Unpaired two-tailed Students’s *t*-test was used for comparisons between two sets of the experiments performed in triplicates for at least three independent times. * denotes significant difference between the two sets at *P*<0.01 and ** at *P*<0.05.

## Abbreviations

*Mtb*: *mycobacterium tuberculosis*
ROS: reactive oxygen species
MOMP: mitochondrial outer membrane potential
UT: untreated/non-activate
Ac: classically activated/IFN-gamma+LPS treated
Nor/NOR: normoxia
Hyp/HYP: hypoxia
GLC: glucose supplementation
OLG: oligomycin treatment.
