# Supplementary material for: Hypoxia and classical activation limits Mycobacterium tuberculosis survival by Akt-dependent glycolytic shift in macrophages
Source: Cell Death Discov. 2016 May 30;2:16022–. doi: 10.1038/cddiscovery.2016.22 (PMC4979487; doi:10.1038/cddiscovery.2016.22)
Supplement: Supplementary Figures [file cddiscovery201622-s1.pdf]

SUPPLEMENTARY INFORMATION

Hypoxia and classical activation limits *Mycobacterium tuberculosis* survival by Akt dependent glycolytic shift in macrophages

Sumit Kumar Matta and Dhiraj Kumar*

Cellular Immunology Group, International Centre for Genetic Engineering and Biotechnology, Aruna Asaf Ali Marg, New Delhi -110067, India

SUPPLEMENTARY FIGURE

Fig. S1

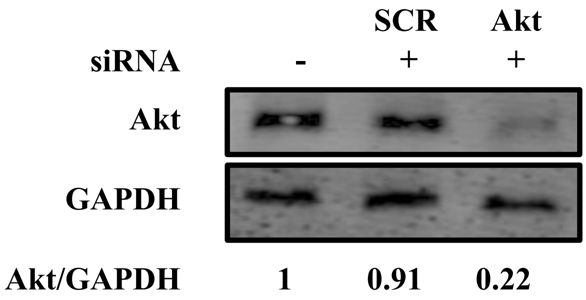

Fig S1. Akt Immunoblot of RAW 264.7 cells treated with 50 nM of scrambled (SCR) and Akt siRNA for 48 hours. GAPDH was used as loading control.

Fig. S2

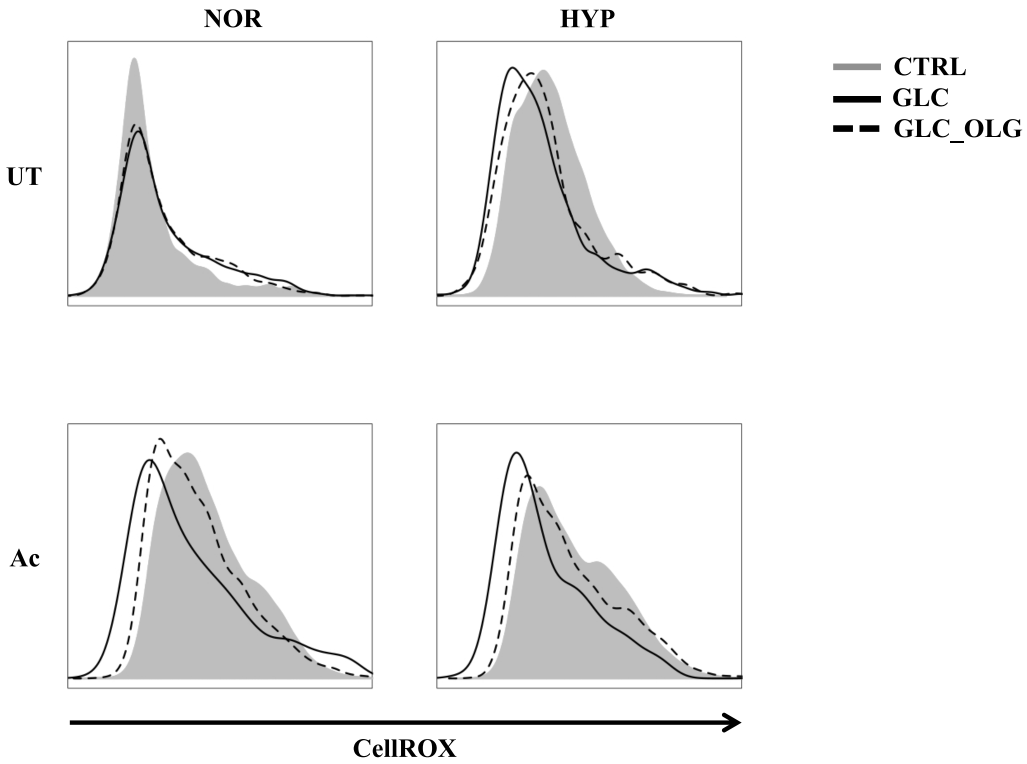

Fig. S2: Line histograms of 10,000 of untreated (UT) and activated (Ac) cells stained with CellROX Green to measure cellular ROS levels. The cells were incubated with solvent control (CTRL) and glucose (10 mM, GLC) without and with oligomycin (200 nM, GLC_OLG) post 24 hours of incubation under normoxia and hypoxia for 48 hours.

Fig. S3

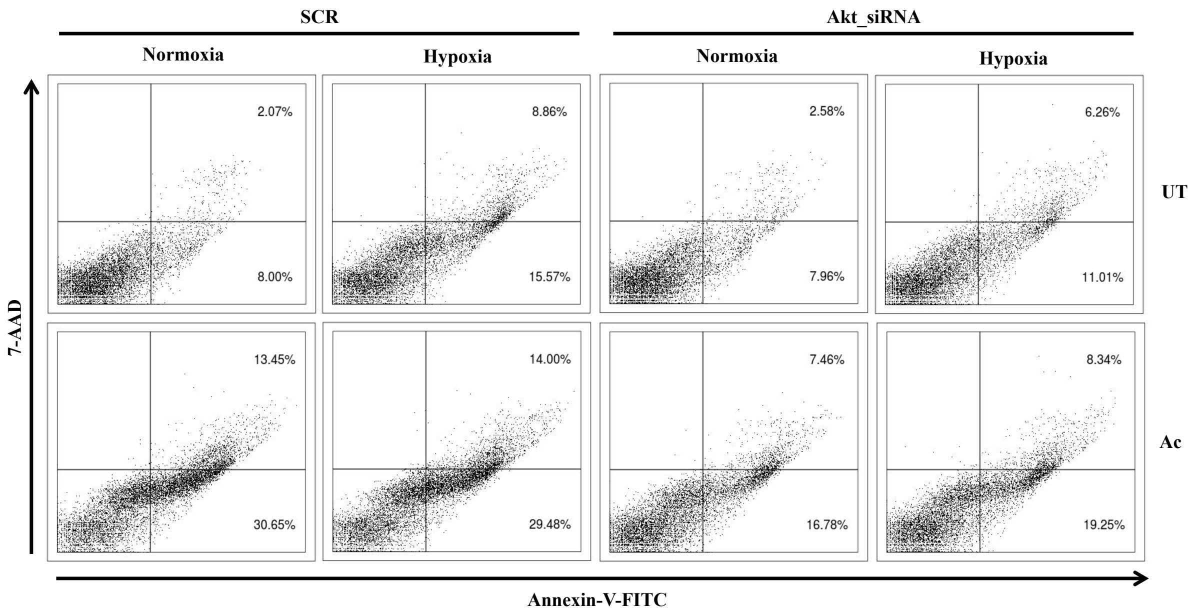

Fig. S3. Scatterplots for Annexin-V FITC and 7-AAD stained untreated (UT) and activated (Ac) cells infected with H37Rv (MOI 10) with respective percentage of cells in their quadrants. The cells were treated along with 50 nM of Scrambled (SCR) and Akt-siRNA under normoxia and hypoxia for 48 hours.
